# A prospective cohort study on the association between dietary fatty acids intake and risk of hypertension incident

**DOI:** 10.1038/s41598-023-48256-5

**Published:** 2023-11-30

**Authors:** Ebrahim Shakiba, Farid Najafi, Yahya Pasdar, Mehdi Moradinazar, Jafar Navabi, Mohammad Hossein Shakiba, Amir Bagheri

**Affiliations:** 1 Research Center for Environmental Determinants of Health (RCEDH), Kermanshah University of Medical Sciences, Kermanshah, Iran; 2 Research Center for Environmental Determinants of Health (RCEDH), Health Institute, Kermanshah University of Medical Sciences, Kermanshah, Iran; 3 Clinical Research Development Center, Imam Khomeini and Mohammad Kermanshahi and Farabi Hospitals, Kermanshah University of Medical Sciences, Kermanshah, Iran; 4 School of Medicine, Kermanshah University of Medical Sciences, Kermanshah, Iran; 5 School of Nutrition Sciences and Food Technology, Kermanshah University of Medical Sciences, Kermanshah, Iran

**Keywords:** Cardiovascular diseases, Nutrition

## Abstract

There are inconclusive results available on the association between dietary fatty acid intake and the risk of hypertension (HTN) incident. In this study, we investigate the relationship between baseline dietary fatty acids intake including polyunsaturated fatty acid (PUFA), trans fatty acids (TFA), monounsaturated fatty acid (MUFA), and saturated fatty acid (SFA), and the risk of first incidence hypertension. The current prospective cohort study was carried out from the Ravansar Non-Communicable Diseases (RaNCD) cohort. A food frequency questionnaire (FFQ) with 118 items was used for the assessment of dietary data. Cox proportional hazards analyses were done to estimate hazard ratios (HR) and 95% confidence intervals (CIs) of the highest versus lowest quartile intake of SFA, PUFA, MUFA, and SFA and risk of HTN. Out of 7359 eligible participants, 597 new cases of HTN were identified over an average of 6.4 ± 1.33 years of follow-up. No significant relationship was observed between the fourth compared to the first categories of dietary SFA (HR: 0.82, 95% CI 0.55, 1.21; P trend: 0.476), MUFA (HR: 0.71, 95% CI 0.48, 1.06; P trend: 0.252), PUFA (HR: 0.86, 95% CI 0.62, 1.19; P trend: 0.315) and TFA (HR: 0.99, 95% CI 0.76, 1.27; *P* trend: 0.675), and risk of HTN. However, a significant inverse association between each 1 g per day increase in dietary MUFA intake during 6.4 years of follow up and HTN incident (HR: 0.97; 95% CI 0.94, 0.99; *P* 0.044) was observed. In brief, our study revealed that higher dietary MUFA intake was protectively associated with HTN incident. Dietary MUFA-rich foods should be encouraged to improve blood pressure.

## Introduction

Hypertension (HTN) is extensively associated with most of chronic disease such as cardiovascular disease (CVD), chronic kidney disease (CKD), end-stage renal disease (ESRD) and mortality^1^. The American Heart Association expected that the direct and indirect costs of HTN in the US population would rise to 240 billion USD by 2030^2^. The prevalence of HTN is lower in high-income countries than in low and middle-income countries^1^. A meta-analysis conducted on Iranian population in 2019, reported that the prevalence of HTN is 22% among adults^3^.

Based on evidence, lifestyle factors like dietary habits, alcohol consumption, and physical inactivity are a main contributor to the high prevalence of this disease^3^. Preventing hypertension through dietary modification is a best solution for public health to promote the population`s well-being^4^. Although the effects of dietary sodium, potassium, fruit, vegetable and obesity on controlling blood pressure are well-established, the roles of dietary fatty acids are ambiguous^5,6^.

The macronutrient known as dietary fat plays a crucial role in providing energy for physical activities, as well as aiding in the transportation of fat-soluble vitamins and the construction of cell membranes^4^. However, overconsumption of dietary fat can lead to weight gain and an increased risk of developing various diseases^4^. Examples of dietary fats include saturated fatty acid (SFA), trans fatty acid (TFA), monounsaturated fatty acid (MUFA), and polyunsaturated fatty acid (PUFA)^4^. A cross-sectional analysis from 18 countries^7^ have revealed that dietary monounsaturated fatty acids (MUFA) intake was inversely related to HTN. Prospective cohort studies have reported mixed results^8–10^. A cohort study on US adults after four years of follow-up did not find any significant associations between dietary SFA or PUFA intake and risk HTN^9^. However, a trial study in the United State during six years of follow up reported that higher dietary intake of PUFA was related to lower blood pressure, while higher dietary SFA intake was associated with higher blood pressure^8^. Another prospective cohort study on 28,100 US women indicated that after considering all potential confounders monounsaturated fatty acids (MUFA), SFA, and PUFA were not associated with risk of HTN, whereas the highest versus lowest quintile of trans fatty acids (TFA) intake was significantly related to developing risk of HTN^10^.

Due to inconclusive results on the association of dietary fatty acids intake and risk of HTN, and also limited study on this relationship specially in developing countries, conducting a cohort study in this regard seems to be necessary. Therefore, we performed a prospective cohort study in the Kurdish adult population of Ravansar Non-Communicable Disease (RaNCD) cohort to determine the link between dietary intake of fatty acids including PUFA, SFA, MUFA, and TFA and the risk of first incidence hypertension.

## Materials and methods

### Study population

**Figure 1:**
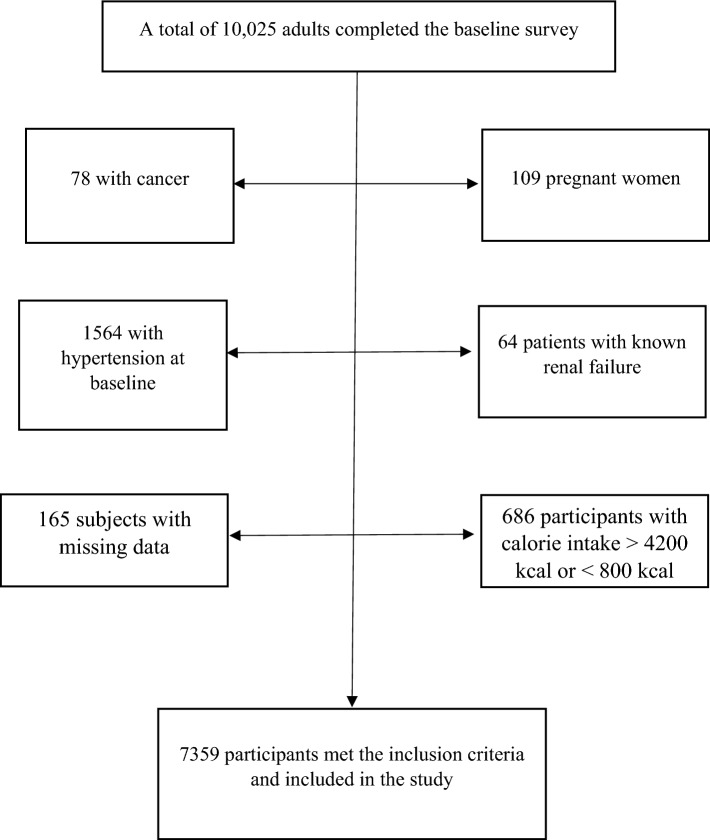
Flowchart of the present study.

This population-based prospective cohort study was carried out from the Ravansar Non-Communicable Diseases (RaNCD) cohort. The inclusive details on the study have been published^11,12^. Overall, RaNCD cohort aims to examine the non-communicable diseases in Iranian Kurdish ethnicity in Ravansar city, Kermanshah Province, west of Iran. Ravansar city with a population of around 50,000 people located in the western part of Iran. The RaNCD cohort was one of 20 cores of the Prospective Epidemiological Research Studies in Iran (PERSIAN) cohort study which is approved by the ethics committees at the Ministry of Health and Medical Education of Iran. Baseline information of 10,025 adults ages of 35 and 65 was collected from October 2014 up to August 2022^11^. Out of 10,025 participants at baseline, 2,666 participants were excluded according to the following reasons: 78 with cancer, 109 pregnant women, 1564 who had hypertension at baseline (prevalence case), 64 patients with known renal failure at baseline, 165 subjects with missing data, and 686 participants with calorie intake higher than 4200 kcal or lower than 800 kcal. Finally, 7359 were eligible for analysis and were included in the study (Fig. 1).

### Assessment of dietary intake

Baseline dietary data of the study participants were collected by 118 items food frequency questioners (FFQ)^11^. This valid and reliable FFQ contains a list of usual and local food with normal service sizes that consumed by Iranian Kurdish population^11^. After fulfilling the FFQ by trained researchers, the reported food item frequency was converted to gram per day by considering the portion sizes of consumed foods^13^. Daily energy, nutrients, MUFA, PUFA, SFA and TFA intakes of each participant was calculated via Nutritionist IV software with a revised food composition dataset for Iranian foods according to the US Department of Agriculture^14^.

### Assessment and definition of blood pressure

Blood pressure of participants was measured by a manometer (Reister) cuff and stethoscope (Reister) while people sit on a chair in relaxed position. The measurement was done from both right and left arm, then it was repeated 10 min later. To reduce the effects of diurnal variation, the blood pressure was measured in the morning. The average of all measurement was considered as the final blood pressure. Finally, based on the codes I10 of the International classification of diseases Tenth Edition (ICD-10), HTN incident was determined. Accordingly, HTN incident was diagnosis as either SBP/DBP ≥ 140/90 mmHg or taking anti-hypertensive medications in the during between baseline of cohort recrutment (2014) and follow up sessions (from 2015 to 2022)^11^.

### Assessment of other factors

Face-to-face interviews and clinical examinations were conducted to examine all participants. Database applications were used to manage data and collect it. For instance, all questionnaires are fulfilled by the online application and controlled by evaluation teams. The Online electronic questionnaire system was used to register the information about living area (urban, rural), marital status, physical activity, age, education, sex, socioeconomic status (SES), smoking history, and history of chronic diseases. All contributors were requested to take off their shoes, heavy dresses, and accessories. Then, hip circumference, waist circumference (WC), and height were measured by skilled investigators. After that, body mass index (BMI) and body weight was calculated by an automated Bio-Impedance Analyzer BIA (Inbody 770, Inbody Co, Seoul, Korea)^11^. To calculate participants’ physical activity, the PERSIAN Cohort standard physical activity questionnaire which consisted of 22 items was applied. Then, participants were categorized into light, moderate, and high according to physical activity intensity. More details on the measurement standard protocols have been published elsewhere^11^.

### Statistical analysis

Dietary fats including SFA, PUFA, MUFA and SFA were adjusted for total energy intake through residual method introduced by Willett^15^. Based in this model, energy-adjusted dietary fats are calculated from the regression model, while the dietary fats considered as a dependent variable and total dietary energy intake as an independent variable. All participants were categorized according to the quartile of energy-adjusted dietary fats and the first quartile were considered as reference. General characteristics of study subjects across the quartile categories of energy-adjusted dietary fats were compared using ANOVA analysis for continuous data and Chi-square analysis for categorical variables. All categorical data are shown as (%), and continuous data are depicted as mean ± standard deviation (SD). To examine the association between SFA, PUFA, MUFA and SFA and risk of HTN incident, Cox proportional hazards analyses were applied to estimate hazard ratios (HR) and 95% confidence intervals (CIs). The time covariate for each subject was defined as the time interval between the baseline entry of cohort data and the incidence of a HTN event. Three cox regression models were defined to adjust for protentional confounders. Model 1 was a crude model. In model 2, we controlled the association for sex (male/female) and age (years). In the third model, we additionally adjusting the association for residence type (rural/urban), marital status, BMI, smoking, SES, history of diabetes, cardiovascular diseases (CVD) history, tertiles of physical activity, tertiles of education, energy intake (kcal/d), sodium (g/d), magnesium (mg/d), carbohydrate (%), protein (%) and healthy eating index-2015 score. Subgroup analyses based on sex and resident type were also performed. All of the statistical analyses were analyzed using SPSS software (version 26). The significance level was set at a *P* value less than 0.05.

### Ethical approval and consent to participate

The Ethics Committee of Kermanshah University of Medical Sciences approved the study (KUMS.REC. 1400.670). All methods were carried out in accordance with relevant guidelines and regulations. The study was conducted in accordance with International Conference on Harmonisation and the ethical principles of the Declaration of Helsinki. All the participants were provided written informed consent.

## Result

Among 7359 participants free of HTN at baseline, a total of 597 (8.11%) new cases of HTN were diagnosed through an average of 6.4 ± 1.33 years of follow-up. Table 1 indicates the baseline characteristics of RaNCD participants by quartiles of dietary TFA, SFA, MUFA, and PUFA intakes. Subjects with the greatest intake of SFA were more likely to be female, residents in rural areas, low SES, educated, and less likely to be a smoker and married. Also, these subjects have lower SBP and DBP. Participants with higher dietary intake of MUFA were more likely to be younger, have lower SBP, female, residents in urban areas, higher SES, and less likely to be educated and physically active. Concerning dietary PUFA intake, subjects consuming higher amounts were younger, and heavier, more likely to be female, urban residents, and higher SES, while less likely to be smokers, physically active, and educated. Participants at the top quartile of TFA intake had younger, lower SBP and DBP, were more likely to be female, urban residents, and higher SES, while less probable to be physically active, educated, smokers, and had CVD or diabetes history.

**Table 1:** General characteristics of study participants in quartile (Q) of dietary fatty acids intakes in RaNCD cohort study.

	SFA			MUFA			PUFA			TFA		
	Q1	Q4	P^1^	Q1	Q4	P^1^	Q1	Q4	P^1^	Q1	Q4	P^1^
Age (Y)	46.2 ± 7.6	46.6 ± 8.1	0.479	46.8 ± 7.7	45.3 ± 7.8	< 0.001	47.7 ± 8.02	44.3 ± 7.2	< 0.001	46.7 ± 7.9	45.3 ± 7.6	< 0.001
BMI	27.2 ± 4.4	27.3 ± 4.7	0.573	27.1 ± 4.5	27.3 ± 4.6	0.657	26.7 ± 4.6	27.8 ± 4.6	< 0.001	27.2 ± 4.5	27.2 ± 4.6	0.967
WHR	0.9 ± 0.06	0.9 ± 0.06	0.184	0.9 ± 0.06	0.9 ± 0.06	0.997	0.9 ± 0.06	0.9 ± 0.06	< 0.001	0.9 ± 0.06	0.9 ± 0.06	0.388
DBP	68.4 ± 7.9	67.5 ± 7.8	< 0.001	68.01 ± 7.8	67.6 ± 7.9	0.081	67.8 ± 7.9	67.4 ± 7.8	0.345	68.3 ± 7.8	67.7 ± 7.9	< 0.001
SBP	105.4 ± 12.5	103.4 ± 12.6	< 0.001	104.6 ± 12.7	103.4 ± 12.3	0.006	104.3 ± 12.8	103.6 ± 12.3	0.148	105.1 ± 12.6	103.8 ± 12.4	0.001
Female (%)	41.1	61.6	< 0.001	45.2	54.9	< 0.001	44.0	58.8	< 0.001	43.1	50.4	< 0.001
Urban-resident (%)	63.3	57.6	< 0.001	49.8	72.8	< 0.001	40.1	82.9	< 0.001	51.6	73.0	< 0.001
Married (%)	92.9	88.3	< 0.001	92.1	89	0.006	91.1	89.4	0.113	91.7	91.2	< 0.001
Current smoker (%)	13.2	10.9	0.009	13.5	12.0	< 0.001	15.0	8.6	< 0.001	13.3	12.5	< 0.001
Low SES (%)	16.6	22.7	< 0.001	23.2	14.2	< 0.001	29.0	9.7	< 0.001	23.1	13.6	< 0.001
High level of Education (%)	38.1	47.8	< 0.001	45.7	38.8	< 0.001	51.3	32.0	< 0.001	43.4	37.2	< 0.001
History of diabetes	6.7	7.2	0.948	7.8	6.6	0.066	6.0	8.2	0.059	6.2	5.7	0.017
History of CVD (%)	6.5	6.3	0.969	5.9	5.8	0.093	5.7	6.0	0.380	5.9	5.3	0.021
Incidence of HTN (%)	8.1	7.6	0.691	8.4	7.0	0.133	8.6	7.7	0.740	7.4	7.3	0.016
High physical activity (%)	22.7	22.8	< 0.001	27.2	19.8	< 0.001	32.5	14.6	< 0.001	27.8	21.7	< 0.001

BMI: Body mass index; WHR: Waist-to-hip ratio; DBP: Diastolic blood pressure; SBP: Systolic blood pressure; SES: Socioeconomic status; CVD: Cardiovascular disease; HTN: Hypertension; SFA: Saturated fatty acid; MUFA: Monounsaturated fatty acid: TFA: Trans fatty acids; PUFA: Polyunsaturated fatty acid.

1: *P*-value attained by ANOVA for continuous variables and chi-square test for categorical variables.

Dietary nutrient intake of the study participants across quartile categories of dietary fatty acids is shown in Table 2. Participants at the highest quartile of SFA intake had a lower intake of energy, carbohydrate, protein, potassium, fiber, and magnesium, and also had a higher intake of dietary fat, sodium, and calcium. Subjects in the top quartile of MUFA intake consume a greater amount of fat and potassium while consuming a lower amount of energy, carbohydrate, protein, fiber, sodium, and magnesium. Dietary intake of energy, carbohydrate, sodium, and calcium declined with increasing intake of PUFA. However, dietary intake of fat, fiber, protein, potassium, and magnesium increased with rising dietary PUFA intake. Concerning dietary TFA intake, participants at the fourth quartile had a lower intake of energy, carbohydrate, fiber, sodium, potassium calcium, and magnesium.

**Table 2:** Dietary nutrients intake of study participants across quartile (Q) categories of dietary fatty acids.

	SFA			MUFA			PUFA			TFA		
	Q1	Q4	P^1^	Q1	Q4	P^1^	Q1	Q4	P^1^	Q1	Q4	P^1^
Energy	2721.9 ± 686.6	2673.8 ± 736.6	< 0.001	2736.2 ± 695.8	2675.2 ± 733.9	< 0.001	2771.2 ± 674.1	2683.9 ± 716.8	< 0.001	3026.1 ± 550.5	2629.1 ± 750.7	< 0.001
Carb (%)	66.9 ± 4.3	54.6 ± 4.7	< 0.001	66.7 ± 4.4	54.8 ± 5.1	< 0.001	64.9 ± 5.2	57.6 ± 5.9	< 0.001	61.5 ± 6.2	60.4 ± 5.9	< 0.001
Fat (%)	21.1 ± 3.5	33.8 ± 4.75	< 0.001	21.1 ± 3.6	33.9 ± 4.8	< 0.001	22.8 ± 4.4	31.1 ± 5.8	< 0.001	26.4 ± 6.1	28.1 ± 5.7	< 0.001
Protein (%)	13.8 ± 2.01	13.4 ± 2.3	< 0.001	13.7 ± 1.8	13.6 ± 2.6	< 0.001	13.6 ± 1.8	13.7 ± 2.3	0.002	13.7 ± 2.1	13.7 ± 2.2	0.304
Fiber (g)	26.1 ± 9.1	21.2 ± 8.1	< 0.001	24.7 ± 8.7	23.0 ± 8.7	< 0.001	23.1 ± 7.9	25.4 ± 8.6	< 0.001	25.5 ± 8.2	24.1 ± 8.6	< 0.001
Sodium (mg)	4519.3 ± 1570.1	5323.7 ± 2036.1	< 0.001	5169.7 ± 1851.9	4631.3 ± 1773.7	< 0.001	5287.2 ± 1833.8	4667.9 ± 1772.2	< 0.001	5627.5 ± 1775.1	4614.5 ± 1756.7	< 0.001
Potassium (mg)	3317.1 ± 1153.6	3067.9 ± 1112.9	< 0.001	3116.1 ± 1123.2	3288.1 ± 1146.5	< 0.001	2975.9 ± 1020.1	3489.2 ± 1120.7	< 0.001	3373.2 ± 1083.9	3287.4 ± 1115.6	< 0.001
Calcium (mg)	1279.8 ± 417.9	1304.8 ± 496.8	< 0.001	1454.2 ± 462.4	1129.4 ± 415.5	< 0.001	1478.6 ± 453.6	1155.1 ± 411.1	< 0.001	1546.8 ± 422.1	1173.6 ± 432.4	< 0.001
Magnesium (mg)	349.6 ± 101.1	299.4 ± 96.9	< 0.001	331.7 ± 95.5	323.2 ± 106.6	< 0.001	318.1 ± 86.9	346.3 ± 107.3	< 0.001	353.7 ± 90.5	328.1 ± 102.7	< 0.001

SFA: Saturated fatty acid; MUFA: Monounsaturated fatty acid: TFA: Trans fatty acids; PUFA: Polyunsaturated fatty acid.

1: *P*-value attained by ANOVA.

HR and 95% CI of HTN according to dietary fatty acids quartile categories are given in Table 3. In model 1 (crude) and model 2 (controlled for age and sex), no significant link was observed between the top vs. bottom quartile of dietary SFA, MUFA, PUFA, and TFA and the risk of HTN. In model 3, after adjusting for all confounders including residence type (rural/urban), marital status, BMI, smoking, SES, history of diabetes, and cardiovascular diseases (CVD), tertiles of physical activity, tertiles of education, energy intake (kcal/d), sodium (g/d), magnesium (mg/d), carbohydrate (%), protein (%) and healthy eating index-2015 score, we did not find any significant link of the fourth compared to the first categories of dietary SFA (HR: 0.82, 95% CI 0.55, 1.21; *P* trend: 0.476), MUFA (HR: 0.71, 95% CI 0.48, 1.06; *P* trend: 0.252), PUFA (HR: 0.86, 95% CI 0.62, 1.19; *P* trend: 0.315) and TFA (HR: 0.99, 95% CI 0.76, 1.27; *P* trend: 0.675), and HTN risk. However, continuous analysis revealed that each gram per day increase in dietary MUFA intake was associated with lower risk of HTN (HR: 0.97; 95% CI 0.94, 0.99; *P*: 0.044).

**Table 3:** Hazard ratio and 95% confidence intervals of hypertension according to quartile categories of dietary fatty acids intake.

	SFA intake						
	Q1	Q2	Q3	Q4	*P* trend	HR for 1 g/day	*P*
Mean SFA intake (g/d)	20.03 ± 7.45	23.49 ± 8.58	29.13 ± 8.77	42.32 ± 12.07			
Number of HTN/number of subjects	149 / 1690	150 / 1690	159 / 1681	139 / 1701			
Crude	1	1.01 (0.80, 1.26)	1.07 (0.85, 1.33)	0.93 (0.74, 1.17)	0.681	0.99 (0.99, 1.01)	0.679
Model 1	1	0.94 (0.75, 1.18)	0.97 (0.78, 1.22)	0.83 (0.66, 1.04)	0.161	0.99 (0.98, 1.01)	0.159
Model 2	1	0.92 (0.72, 1.17)	0.97 (0.73, 1.29)	0.82 (0.55, 1.21)	0.476	0.99 (0.97, 1.01)	0.451

	MUFA intake						
	Q1	Q2	Q3	Q4	*P* trend	HR for 1 g/day	P
Mean **MUFA** intake (g/d)	13.61 ± 4.98	16.32 ± 5.51	20.05 ± 5.62	29.48 ± 8.35			
Number of HTN/number of subjects	158 / 1681	153 / 1687	144 / 1696	142 / 1698			
Crude	1	0.94 (0.75, 1.18)	1.07 (0.86, 1.33)	0.81 (0.64, 1.02)	0.207	0.99 (0.97, 1.01)	0.130
Model 1	1	0.88 (0.70, 1.11)	1.03 (0.83, 1.29)	0.85 (0.67, 1.07)	0.421	0.99 (0.98, 1.01)	0.318
Model 2	1	0.84 (0.65, 1.07)	0.94 (0.71, 1.24)	0.71 (0.48, 1.06)	0.252	0.97 (0.94, 0.99)	0.044

	PUFA intake						
	Q1	Q2	Q3	Q4	*P* trend	HR for 1 g/day	P
Mean PUFA intake (g/d)	7.36 ± 2.69	8.73 ± 3.09	11.15 ± 3.39	17.32 ± 5.17			
Number of HTN/number of subjects	158 / 1681	153 / 1687	144 / 1696	142 / 1698			
Crude	1	0.96 (0.77, 1.20)	0.90 (0.71, 1.12)	0.86 (0.70, 1.11)	0.237	0.98 (0.96, 1.01)	0.135
Model 1	1	0.93 (0.74, 1.17)	0.92 (0.73, 1.15)	1.02 (0.81, 1.29)	0.890	0.99 (0.97, 1.02)	0.866
Model 2	1	0.87 (0.68, 1.10)	0.80 (0.61, 1.05)	0.86 (0.62, 1.19)	0.315	0.97 (0.95, 1.01)	0.175

	TFA intake						
	Q1	Q2	Q3	Q4	P trend	HR for 1 g/day	P
Mean TFA intake (g/d)	0.14 ± 0.07	0.16 ± 0.09	0.20 ± 0.11	0.64 ± 0.50			
Number of HTN/number of subjects	136 / 1703	146 / 1694	181 / 1659	134 / 1706			
Crude	1	1.07 (0.85, 1.35)	1.34 (1.08, 1.68)	0.98 (0.77, 1.24)	0.613	0.92 (0.71, 1.19)	0.526
Model 1	1	1.01 (0.79, 1.27)	1.23 (0.98, 1.54)	1.04 (0.81, 1.32)	0.352	1.01 (0.78, 1.32)	0.891
Model 2	1	0.97 (0.75, 1.24)	1.19 (0.92, 1.54)	0.99 (0.76, 1.27)	0.675	0.98 (0.74, 1.29)	0.894

SFA: Saturated fatty acid; MUFA: Monounsaturated fatty acid: TFA: Trans fatty acids; PUFA: Polyunsaturated fatty acid.

Model 1: Adjusted for age and sex.

Model 2: Adjusted for model 1 + Residence Type, Marital Status, BMI, Smoking, SES, Diabetes, cardiovascular diseases, physical activity, Education, Energy intake, Sodium, Healthy eating index-2015, Percent of carbohydrate, Percent of protein, and Magnesium.

Subgroup analyses for sex and resident type by dietary fatty acids intake and risk hypertension are depicted in Table 4. No significant relationship was observed between dietary SFA, MUFA, PUFA, and TFA intakes and HTN risk across subgroups of sex and resident type.

**Table 4:** Subgroup analyses for dietary fatty acids intake and hypertension.

	SFA intake			
	Multivariable HR and 95% CI for Highest vs. lowest *	*P* trend	HR for 1 g/day	*P*
Sex				
Men	0.56 (0.28, 1.09)	0.05	0.99 (0.96, 1.02)	0.532
Women	1.09 (0.67, 1.79)	0.43	0.99 (0.97, 1.02)	0.851
Resident type				
Urban	0.72 (0.45, 1.17)	0.216	0.98 (0.96, 1.01)	0.065
Rural	1.09 (0.55, 2.14)	0.592	1.02 (0.99, 1.05)	0.163

	MUFA intake			
	Multivariable HR and 95% CI for Highest vs. lowest	*P* trend	HR for 1 g/day	*P*
Sex				
Men	0.60 (0.31, 1.15)	0.161	0.96 (0.92, 1.01)	0.135
Women	0.90 (0.53, 1.51)	0.993	0.98 (0.95, 1.02)	0.403
Resident type				
Urban	0.80 (0.49, 1.31)	0.512	0.97 (0.94, 1.01)	0.164
Rural	0.55 (0.26, 1.13)	0.275	0.96 (0.92, 1.01)	0.092

	PUFA intake			
	Multivariable HR and 95% CI for Highest vs. lowest	*P* trend	HR for 1 g/day	*P*
Sex				
Men	0.81 (0.48, 1.37)	0.191	0.99 (0.94, 1.04)	0.688
Women	0.96 (0.63, 1.44)	0.950	0.97 (0.94, 1.02)	0.262
Resident type				
Urban	0.91 (0.61, 1.35)	0.598	0.97 (0.94, 1.01)	0.235
Rural	0.65 (0.34, 1.24)	0.238	0.97 (0.92, 1.04)	0.455

	TFA intake			
	Multivariable HR and 95% CI for Highest vs. lowest	*P* trend	HR for 1 g/day	*P*
Sex				
Men	0.94 (0.64, 1.38)	0.926	0.97 (0.63, 1.50)	0.920
Women	1.09 (0.77, 1.54)	0.436	1.03 (0.72, 1.47)	0.879
Resident type				
Urban	0.97 (0.71, 1.33)	0.555	0.99 (0.73, 1.35)	0.968
Rural	0.97 (0.61, 1.54)	0.828	0.86 (0.44, 1.67)	0.663

SFA: Saturated fatty acid; MUFA: Monounsaturated fatty acid: TFA: Trans fatty acids; PUFA: Polyunsaturated fatty acid.

*Adjusted for model 1 + Residence Type, Marital Status, BMI, Smoking, SES, Diabetes, cardiovascular diseases, physical activity, Education, Energy intake, Sodium, Healthy eating index-2015, Percent of carbohydrate, Percent of protein, and Magnesium.

## Discussion

The current prospective cohort study on Iranian Kurdish adults revealed that higher dietary MUFA intake was protectively associated with HTN risk, while dietary SFA, TFA, and PUFA intakes were not related to the risk of HTN. The associations were independent of age, gender, residence type, marital status, BMI, smoking, SES, history of diabetes and CVD, physical activity, education, energy intake, sodium, magnesium, carbohydrate, protein and healthy eating index-2015 score. As far as we know, there is a limited cohort study available on the association between dietary fatty acid intake and HTN in the middle east countries.

A survey in 2010 estimated that out of 1.39 billion adults with HTN worldwide, 1.04 billion live in low and middle-income countries^16^. Higher blood pressure is strongly and independently associated with the risk of CVD, ESRD, and mortality^1^. Therefore, reductions in HTN risk factors are suggested for the prevention and control of this disease^1^. In our study, dietary MUFA intake was protectively related to HTN incidence, whereas dietary SFA, TFA, and PUFA intakes did not significantly relate to HTN. In line with the findings, a randomized controlled parallel trial in five European countries indicated that SBP and DBP were significantly decreased by following a high MUFA diet but did not change by adherence to a high SFA diet^17^. Moreover, two other RCTs in a population at risk of CVD showed high MUFA diet compared to a high carbohydrate diet could significantly reduce blood pressure^18,19^. A cross-sectional survey in the USA revealed that after controlling for all protentional covariates dietary MUFA intake was inversely associated with systolic and diastolic blood pressure^20^. Similar to our results, two prospective cohort studies on US men^9^ and women^21^ adults after four years of follow-up did not find any significant relationships between dietary SFA or PUFA intake and HTN risk. In the same way, a cross-sectional analysis conducted on 18 countries in North America, Asia, Europe, South America, and Africa showed that dietary MUFA intake was inversely related to the odds of HTN, while dietary SFA intake was not associated with HTN^7^. In contrast to our results, in a prospective cohort study on 11 342 US adults, PUFA intake was inversely related to blood pressure, while SFA intake was positively associated with blood pressure^8^. Finding from another prospective cohort study on 28,100 US women, indicated that TFA intake was significantly related to developing risk of HTN, while MUFA, SFA, and PUFA intakes were not associated^10^. It is worth noting that the inconsistency in the association between dietary fatty acids intake and hypertension risk could be attributed to variations in the study region, the demographics of the population (age, gender), the type of dietary questionnaire, and the consideration of the energy adjustment of fatty acid intake. Although studies reported mixed results, it seems dietary MUFA intake might have a protective association with HTN.

The beneficial effect of MUFA intake on blood pressure might be due to improvement on the coronary vascular function by increased high-density lipoprotein (HDL) and reduced low-density lipoprotein (LDL) and^22^. Second plausible mechanism is that MUFA by decreasing the inflammatory markers, atherosclerotic lesions, levels of non-esterified fatty acids and trimethylamine-N-oxide level could improve endothelial function^23^. Insulin sensitivity improvement is another suggested mechanism of MUFA which may reduce sympathetic nervous system, sodium retention, and peripheral resistance^24,25^.

Strengths of the current study included the large population in the middle-east country and detailed information over 6.4 years follow up. However, some limitations are unavoidable. First, although we adjusted dietary intake for total energy, measurement errors may still be probable for the dietary assessment. Second, residual confounding factors likely remained even after controlling the association for all covariates. Because our study conducted on Iranian Kurdish ethnicity, generalizing our results to other populations with different demographic characteristics should be done with caution.

## Conclusions

In conclusion, our findings demonstrated that higher dietary MUFA intake was protectively related with HTN risk. Other dietary fatty acids including PUFA, SFA, and TFA were not related to HTN incident. Dietary MUFA rich foods such as canola oil, peanut oil, nuts, olive oil, vegetables, and fruits should be encouraged to improve blood pressure.

## Data Availability

All data generated and analyzed during this study are included in the manuscript.

## Abbreviations

BMI: Body mass index
WHR: Waist-to-hip ratio
DBP: Diastolic blood pressure
SBP: Systolic blood pressure
SES: Socioeconomic status
CVD: Cardiovascular disease
HTN: Hypertension
SFA: Saturated fatty acid
MUFA: Monounsaturated fatty acid
TFA: Trans fatty acids
PUFA: Polyunsaturated fatty acid
